# Global Transcriptional Profiles of the Copper Responses in the Cyanobacterium *Synechocystis* sp. PCC 6803

**DOI:** 10.1371/journal.pone.0108912

**Published:** 2014-09-30

**Authors:** Joaquin Giner-Lamia, Luis López-Maury, Francisco J. Florencio

**Affiliations:** Instituto de Bioquímica Vegetal y Fotosíntesis, Universidad de Sevilla-CSIC, Sevilla, Spain; Belgian Nuclear Research Centre SCK•CEN, Belgium

## Abstract

Copper is an essential element involved in fundamental processes like respiration and photosynthesis. However, it becomes toxic at high concentration, which has forced organisms to control its cellular concentration. We have recently described a copper resistance system in the cyanobacterium *Synechocystis* sp. PCC 6803, which is mediated by the two-component system, CopRS, a RND metal transport system, CopBAC and a protein of unknown function, CopM. Here, we report the transcriptional responses to copper additions at non-toxic (0.3 µM) and toxic concentrations (3 µM) in the wild type and in the copper sensitive *copR* mutant strain. While 0.3 µM copper slightly stimulated metabolism and promoted the exchange between cytochrome c_6_ and plastocyanin as soluble electron carriers, the addition of 3 µM copper catalyzed the formation of ROS, led to a general stress response and induced expression of Fe-S cluster biogenesis genes. According to this, a double mutant strain *copRsufR*, which expresses constitutively the *sufBCDS* operon, tolerated higher copper concentration than the *copR* mutant strain, suggesting that Fe-S clusters are direct targets of copper toxicity in *Synechocystis*. In addition we have also demonstrated that InrS, a nickel binding transcriptional repressor that belong to the CsoR family of transcriptional factor, was involved in heavy metal homeostasis, including copper, in *Synechocystis*. Finally, global gene expression analysis of the *copR* mutant strain suggested that CopRS only controls the expression of *copMRS* and *copBAC* operons in response to copper.

## Introduction

Copper is an essential oligoelement that is required as a cofactor for a number of cuproenzymes including amine oxidases, cytochrome c oxidases, laccases, methane monooxygenases, multicopper oxidases, nitrite oxidases, plastocyanin, superoxide dismutases and tyrosinases. These proteins are involved in diverse cellular processes such as energy transduction, iron mobilization and oxidative stress response [1], [2]. The ability of copper to alternate between its cuprous Cu(I) and cupric Cu(II) oxidation states makes it an ideal biological cofactor. However, the two-oxidation states of copper not only allow its participation in essential redox reactions but also to catalyze the production of reactive oxygen species (ROS) through the Fenton and Haber-Weis reactions, which leads to severe damage to lipids, proteins, DNA and other cytoplasmic molecules [3]. Furthermore, copper in excess competes with other metals for their binding sites in proteins following the Irwing-Williams series [4], resulting in a perturbation of protein function and in some cases protein degradation. Recently, an alternative copper toxicity mechanism has been reported in *Escherichia coli*, *Bacillus subtilis* and *Synechocystis* sp. PCC 6803 (hereafter *Synechocystis*), in which Cu(I), the predominant intracellular species [5], interferes with the function and/or stability of catalytic Fe-S clusters, damaging essential enzymes [6], [7], [8]. All these have forced all living organisms to develop homeostatic mechanisms to tightly control cellular copper pools.

To cope with hazardous copper concentrations, bacteria use copper specific induced mechanisms that include membrane transporters, copper chaperones and copper responsive transcriptional factors. Active efflux is a key feature for copper resistance and three non-related families of export system have been characterized: P_I_-type ATPases, which hydrolyses ATP to drive Cu cations from cytosol to the periplasmic space, like *Escherichia coli* CopA [9], heavy metals efflux-resistance nodulation and division (HME-RND) efflux systems, such as *copBAC* [10], and other membrane proteins, like CopB and CopD from *Pseudomonas syringae* [3], [11]. Copper homeostasis systems usually contain periplasmic and/or cytosolic copper-binding proteins to avoid deleterious side reactions and to ensure that copper is properly delivered to the correct target proteins [12], such as the periplasmic copper chaperone CusF and the cytoplasmic copper chaperones Atx1 or CopZ [3], [13]. Multicopper oxidases are also involved in copper resistance, since they oxidize Cu(I) to Cu(II) in the periplasm, which is a less toxic form that is not transported inside the cell [14], [15]. Copper resistance systems are usually transcriptionally regulated by copper and this regulation is mediated by two types of metalloregulatory proteins systems: copper-responsive transcription factors that sense cytosolic copper levels and belong to several unrelated families of transcriptional regulators, including CueR, CopY, CsoR or BxmR [16], [17], [18], [19], [20], and two-component copper-responsive systems that detect periplasmic copper levels, which the best characterized member is CopRS in *E. coli* [3], [11], [21], [22], [23].

Cyanobacteria are unusual among bacteria as they have internal copper requirements for two proteins: the blue-copper protein plastocyanin and the caa_3_-type cytochrome oxidase which are involved in the photosynthetic and respiratory electron transport chains, respectively. These two proteins are localized in an internal membranous system, the thylakoids. Thus, cyanobacteria constitute an attractive model to investigate the systems managing copper use as a metabolite and those systems used to avoid its toxic effects. In cyanobacteria, copper metabolism has been mainly studied in the model cyanobacterium *Synechocystis*. Copper import in *Synechocystis* is mediated by two P_I_-type ATPases, CtaA and PacS, which are located in the plasma and thylakoidal membranes respectively, a small cytosolic soluble copper metallochaperone, Atx1, and glutathione [8], [24], [25]. Copper import inside the cell is mediated by CtaA, which delivers it to Atx1, that together with glutathione buffers cytoplasmic copper [8], this is subsequently transferred to PacS, which finally transports it into the thylakoid lumen. We have recently described a copper resistance mechanism in *Synechocystis* that comprises a two-component system, CopRS, an HME-RND export system, CopBAC, and a protein of unknown function, CopM [21]. These proteins are encoded by two operons: *copMRS* (which is duplicated in the plasmid pSYSX and designated as *copM_1_R_1_S_1_* and *copM_2_R_2_S_2_* here), and *copBAC*, which is only present in the plasmid pSYSX. The expression of both copies of *copMRS* and *copBAC* is regulated by CopRS in response to the presence of copper in the media [21]. However, CopRS does not control the expression of any of the copper metabolism genes described above, *ctaA*, *pacS* and *atx1* [21]. Mutants in *copRS* (lacking both copies of one of these genes) or *copBAC* render cells more sensitive to copper and accumulate higher amount of copper than the wild type. Moreover, CopS the histidine kinase that detects copper, belongs to the membrane attached histidine kinases and contains a periplasmic domain that presents high copper affinity. Furthermore, CopS is localized not only in the plasma membrane but also in the thylakoid membrane and is involved in copper detection in both the periplasm and the thylakoid lumen [21]. The CopRS is also known as the Hik31-Rre34 two-component system which has been suggested to be implicated in cell growth under mixotrophic and heterotrophic conditions [26], [27], under light dark transitions [28] and also in the regulation of the response to low-oxygen conditions [29].

Here we present the global transcriptional profiles of WT *Synechocystis* and a *copR* mutant strain, COP4, exposed to non-inhibitory (0.3 µM) and inhibitory (3 µM) copper concentrations. The low copper treatment up-regulated expression of genes related to anabolic metabolism while the high copper treatment induced the formation of ROS in the WT strain and leads to a general stress response in both WT and COP4 strains. In addition, analysis of the COP4 strain showed that *copMRS* and *copBAC* are the only genes directly regulated by the CopRS two-component system in response to copper, beyond plasmid genes, which were not analysed in this work. Finally, we showed that the higher copper treatment induced the *suf* system for Fe-S cluster assembly and many other genes related to metal homeostasis. Using different mutants we show that these two processes are essential during copper stress.

## Results and Discussion

### Transcriptional profiles of *Synechocystis* in response to low and high copper treatments

**Figure 1:**
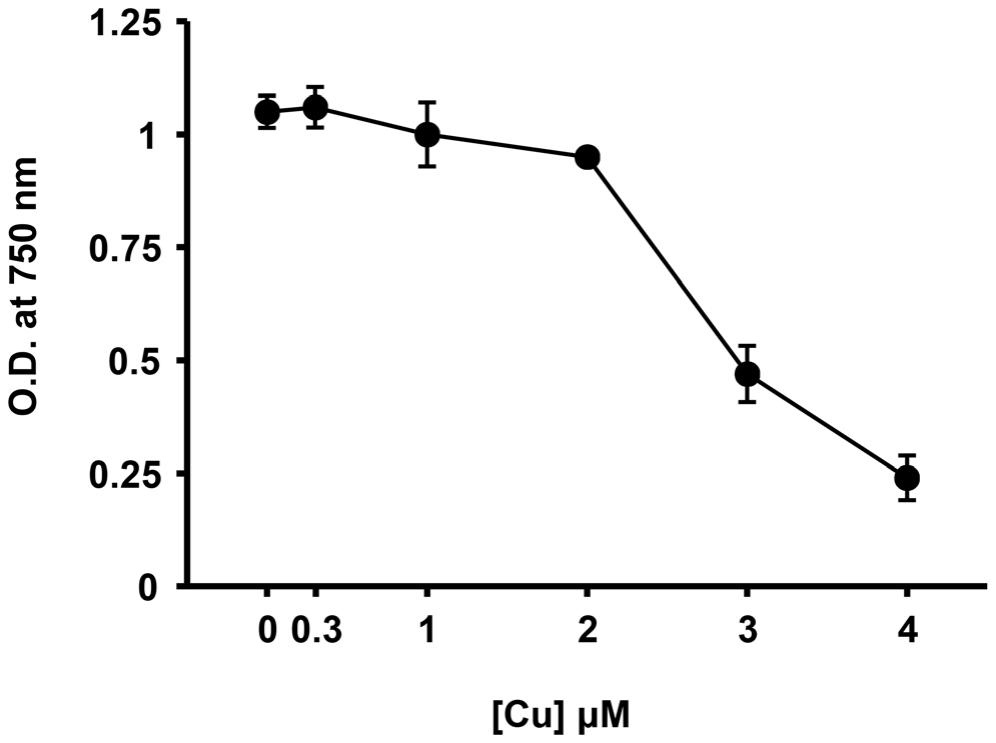
Determination of the minimal inhibitory concentration for copper in *Synechocystis*. Exponentially growing cells of *Synechocystis* WT strain were diluted to OD_750 nm_ of 0.6 and cultured in BG11-Cu medium supplemented with the indicated copper concentration for 24 hours.

In order to establish the appropriate copper concentration for the transcriptional profiling we determined the minimal inhibitory concentration (MIC) for copper. For this purpose, exponentially growing cultures (OD_750 nm_ of 0.6) in BG11C-Cu were treated with different copper concentrations and their growth was monitored after 24 h. *Synechocystis* growth was un affected up to 2 µM copper, whereas the MIC (after 24 h of exposure) was 3 µM (Fig. 1). According to this, we selected 0.3 µM (the concentration present in the standard BG11C and therefore a non-inhibitory concentration) and 3 µM (the MIC in our conditions; Fig. 1) for our microarray transcriptional study. *Synechocystis* cells were grown in BG11C lacking added copper (BG11C-Cu, which has been shown to be a non stressful condition [24], [30]) and 0.3 µM (the standard copper concentration present in BG11C; [31]) or 3 µM CuSO_4_ (the MIC in our conditions; Fig. 1) were added. After 1 hour treatment, RNA was extracted from these samples and used to hybridize one-color Agilent microarrays covering all chromosomal *Synechocystis* genes. Two biological replicates for each copper concentration and four for the control condition (-Cu) were performed. All conditions showed high levels of correlation between separate chip hybridizations (R^2^ = 0.966 for –Cu, R^2^ = 0.984 for low Cu and R^2^ = 0.958 for high Cu samples). In order to identify differentially expressed genes the statistic test Limma was used and genes were considered differentially expressed if they had a fold change ≥2.5 and the *p*<0.01.

### Low copper treatment produces a slight increase in *Synechocystis* metabolism

**Figure 2:**
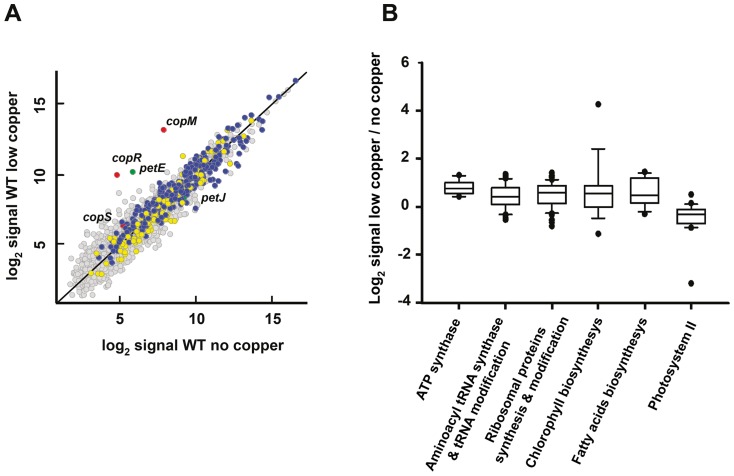
Global responses to low copper treatment in *Synechocystis*. A. Scatter plot showing comparison between expression profiles of WT treated with copper 0.3 µM for 1 h (y axis) and untreated WT (x axis). Data represents the average signal of two independent hybridizations. CTR up-regulated genes are colored in yellow, CTR down-regulated genes in blue, *copMRS* operon in red and the genes for soluble electron carriers *petE* and *petJ* in green. B. Box plot showing ratios of gene list of treated vs. untreated samples of the categories cited in the main text that were significantly affected.

Based on the above explained criteria only 46 genes were differentially expressed after the 0.3 µM Cu treatment, which represent less than 1% of the protein-coding *Synechocystis* genes. Of these, 17 genes were up-regulated and 29 genes were down-regulated (Fig. 2A; Table S1). These genes did not belong to a specific cyanobase category. In order to identify the processes and pathways involved in the transcriptional response to low copper the statistical tool Gene Set Enrichment Analysis (GSEA; [32]) was used. GSEA compares the averages expression of genes within a category and determine if this group is differentially expressed. We applied this method to our expression data using gene functions as defined in the Cyanobase, GO annotation and hand curated gene lists (see material and methods section), which contains gene lists extracted from the literature. The gene lists that were significantly enriched are shown in Table S2. This analysis revealed that after low copper treatment, gene lists containing genes coding for ribosomal proteins, aminoacyl tRNA synthetases, ATP synthetase, biosynthesis of heme groups (including chlorophyll biosynthesis), and fatty acids biosynthetic processes were slightly but significantly up-regulated (Fig. 2B; Table S2), while the only gene list down-regulated cointained genes coding for the Photosystem II (PSII).

Copper is an essential trace element for *Synechocystis* and is used as a cofactor of two proteins involved in energy production: plastocyanin and cytochrome c oxidase. The genes encoding the aa_3_-type cytochrome oxidase c (*ctaCIDIEI*) were not differentially regulated after the low copper treatment. In contrast, the *petE* gene was highly induced although the *petJ* gene was not fully repressed, showing a partial switch between plastocyanin (*petE*) and cytochrome c_6_ (*petJ*) genes (Fig. 2A, Table S1), as previously described for this copper concentration and verified in our condition (Fig. S1; [33]). The increase in plastocyanin expression together with down-regulation of PSII genes suggests an increase in cyclic electron transport and/or respiratory electron transport. This will probably enhance ATP synthesis necessary to deal with an increase in the anabolic metabolism. In fact, a minimal amount of copper is strictly necessary for respiration and heterotrophic growth [30]. Remarkably, we have not observed changes in the expression pattern of genes coding for the copper import system in *Synechocystis* (*ctaA*, *pacS*, *atx1* and *gshB*), indicating that at this copper concentration (or lower) can be managed by the steady state level of these proteins in the cell.

Copper responsive transcriptional factors are able to detect copper at very low concentrations. This is the case of CueR, the copper sensing cytoplasmic transcriptional factor in *Escherichia coli* that is able to respond to copper at a concentration corresponding to less than a free atom per cell [34]. In the case of *Synechocystis*, we have previously determined an apparent affinity of CopS histidine kinase for copper to be 10^−19^ M^−1^ [21] and the analysis of the microarray data is consistent with this data. *copM* and *copR* were the most induced genes by this treatment (52 fold for *copM* and 32 fold for *copR*) suggesting that CopS is activated under this conditions and which shows a strong polar effect in the expression levels of *copMRS* operon (Fig. 2A; Table S1); unfortunately we can not distinguish between the two copies of these genes because of their high level of identity (>93% at nucleotide level) and we will refer to them simply as *copMRS* when analyzing gene expression. This observation also agrees with the fact that CopRS responds to copper released from plastocyanin degradation, in conditions that alter the photosynthetic electron flow, when cells were growing at this copper concentrations [21], [35]. Although this induction is transitory and decreases 4 h after copper addition [21], *copM* is also expressed when cells are cultured in BG11C containing copper (Fig. S2). These data suggest that the *copMRS* system is required, at least transiently, even at concentrations in which copper acts as a micronutrient, probably to prevent any deleterious side effects.

### The high copper treatment induces a general stress response in *Synechocystis*

**Figure 3:**
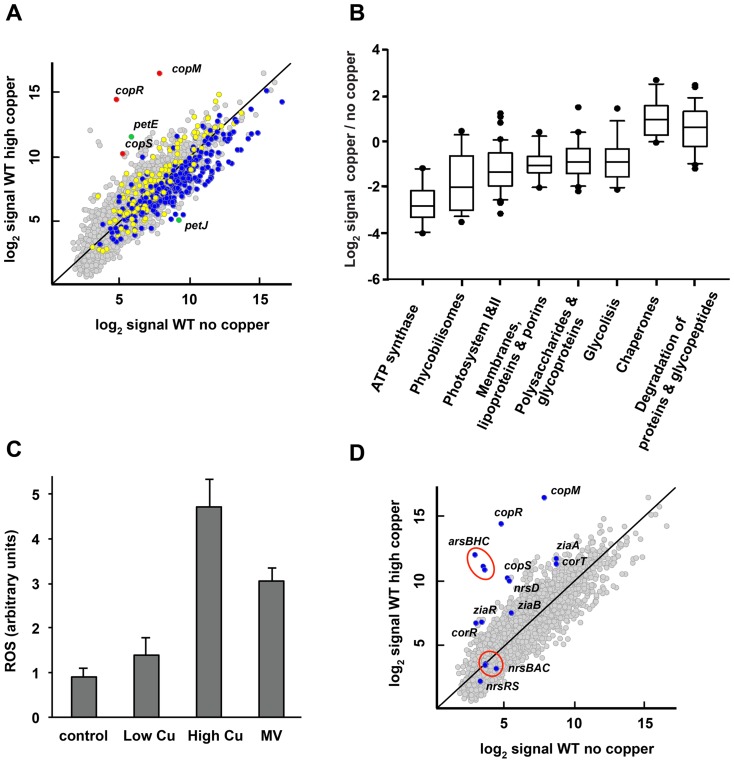
Global responses to high copper treatment in *Synechocystis*. A. Scatter plot showing comparison between expression profiles of WT treated with copper 3 µM for 1 h (y axis) and untreated WT (x axis). Data represents the average signal of two independent hybridizations. CTR up-regulated genes are in yellow, CTR down-regulated genes in blue, *copMRS* operon in red and the genes for soluble electron carriers *petE* and *petJ* in green. B. Box plot showing ratios of gene list of treated vs. untreated samples of the categories cited in the main text that were significantly affected. C. Induction of ROS in response to copper. The determination of ROS in WT cells cultured in BG11C-Cu medium supplemented with 0.3 µM Cu (Low Cu), 3 µM Cu (High Cu) and 5 µM of methyl viologen (MV) for 1 h were determined. Untreated cells were used as control. Values are the mean of three independent experiments. Error bars represent standard error. D. Scatter plot showing comparison between expression profiles of WT treated with copper 3 µM for 1 h (y axis) and untreated WT (x axis). Data represents the average signal of two independent hybridizations. Genes related to heavy metal resistance are shown in blue.

After the high copper treatment (3 µM) 394 genes (12.9% of the protein-coding genes; Table S3) were differentially expressed. Of these, 223 genes were up-regulated and 171 genes down-regulated, showing a drastic response compared to the low copper treatment (Fig. 3A). Although most of these genes belong to unknown function (188 genes) or other processes categories (59 genes) according to cyanobase. In addition, there were several genes classified in the photosynthesis (43 genes), transport and binding protein (29 genes), transcription and translation (22 genes), amino acids biosynthesis (15 genes), redox response and protein misfolding (20 genes) and regulatory function (15 genes) categories (Table S3). GSEA analysis showed enrichment of gene lists that are induced or repressed in other stresses like cadmium [36], high light [37], heat shock [38], H_2_O_2_ treatment [39] or sulphur [40] and nitrogen deprivation [41], reinforcing the idea that a general stress response was triggered after high copper treatment (Table S4). In fact, and in contrast to what happened in the low copper treatment, the expression pattern after the high copper treatment correlates with a general stress response in *Synechocystis* (Fig. 3A). This response mainly consists in the repression of genes related to energy generation and growth processes and the induction of genes sets related to stress like chaperones, proteases or ROS detoxification systems, which has been previously named as Core Transcriptional Response (CTR; Figs. 2A and 3A [42]). Furthermore and according to this, more than 31% of all photosynthetic and respiratory genes in *Synechocystis* were down regulated (Tables 1 and S3), mainly ATP synthesis, PSI, PSII and phycobilisome genes (Fig. 3B). The down-regulation of PSI and PSII genes under high copper conditions has been previously reported in other photosynthetic microorganisms including two strains of *Synechococcus* [43] and in the green alga *Chlamydomonas reindhartii* [44]. An immediate consequence of the repression of these genes is a depletion of the final products from the light reactions and energy production, which eventually affects the CO_2_ fixation and carbon metabolism. In agreement with this down-regulation of *rbcS* and *rbcL* (encoding the two RuBIsCO subunits), *glgP (slr1367*; encoding glycogen phosphorylase), *glgX* (*slr1857*; encoding glycogen isoamylase) and the gene list related to glycolysis was observed (Fig. 3B, Tables 1, S3 and S4). This response was coordinated with a down-regulation of genes involved in nitrogen assimilation similarly to what it has been reported in other stresses [42], [45]. Genes encoding for glutamine synthetase (*glnA* [46]), signal transduction protein PII (*glnB* [47]), and high activity uptake ammonium permease(*amt1* [48]) were down regulated in response to copper (Table 1 and S3). As a consequence of this decrease in carbon and nitrogen assimilation, other growth-related processes were also down regulated, including genes related to transcription (*sigD, sigE, sigH*), translation (*rplR, rpsE, rplF, rplJ, rplL, rbp3*) and amino acids synthesis (*thrA, proA, thrB, ilvD, argC, norB*; Table S3).

**Table 1:** Selected genes repressed in *Synechocystis* sp. PCC 6803 after the high copper treatment.

Locus	Gene	Ratio (3 µM/-Cu)	Description
**Carbon and nitrogen metabolism**			
*slr1367*	*glgP*	0.35	Glycogen phosphorylase.
*slr1857*	*glgX*	0.33	Glycogen isoamylase
*ssl0707*	*glnB*	0.33	Nitrogen regulatory protein PII
*slr1529*	*ntrX*	0.29	Nitrogen assimilation regulatory protein.
*slr1756*	*glnA*	0.26	Glutamine synthetase
*slr0009*	*rbcL*	0.15	RuBisCO large subunit
*slr0012*	*rbcS*	0.10	RuBisCO small subunit
***Transcription***			
*slr1545*	*sigG*	0.34	RNA polymerase sigma-E factor
*sll0856*	*sigH*	0.30	RNA polymerase sigma-E factor
*sll2012*	*sigD*	0.18	RNA polymerase sigma factor SigD
***Translation***			
*sll1811*	*rplR*	0.37	50S ribosomal protein L18
*sll1812*	*rpsE*	0.36	30S ribosomal protein S5
*sll1810*	*rplF*	0.33	50S ribosomal protein L6
*slr0193*	*rbp3*	0.33	RNA-binding protein
*sll1745*	*rplJ*	0.29	50S ribosomal protein L10
*sll1746*	*rplL*	0.28	50S ribosomal protein L12
**Amino acid biosynthesis**			
*sll0455*	*thrA*	0.37	Homoserine dehydrogenase
*sll0461*	*proA*	0.35	Gamma-glutamyl phosphate reductase
*sll1760*	*thrB*	0.31	Homoserine kinase
*slr0452*	*ilvD*	0.29	Dihydroxyacid dehydratase
*sll0080*	*argC*	0.28	N-acetyl-gamma-glutamyl-phosphate reductase
*sll0450*	*norB*	0.12	Cytochrome b subunit of nitric oxide reductase
***Transport and binding proteins***			
*sll0064*		0.18	Putative polar amino acid transport system
*sll1270*	*bgtB*	0.29	ABC-type Bgt permease for basic amino acids and glutamine
*slr0415*	*napA*	0.29	Na+/H+ antiporter
*slr0369*	*envD*	0.28	RND multidrug efflux transporter
*slr0875*	*mscL*	0.25	Large-conductance mechanosensitive channel
*slr0096*		0.24	Low affinity sulfate transporter
*sll1406*	*fhuA*	0.24	Ferrichrome-iron receptor
*sll0224*		0.23	Amino-acid ABC transporter binding protein
*slr1727*		0.22	Na+/H+ antiporter
*sll1087*		0.19	Similar to sodium/glucose cotransporter
*sll0108*	*amt1*	0.15	Ammonium/methylammonium permease
*slr0447*	*urtA*	0.13	ABC-type urea transport system
*sll1206*	*iutA*	0.13	Ferric aerobactin receptor, FhuA homolog
*slr2057*	*apqZ*	0.09	Water channel protein
***Photosynthesis***			
*sll1796*	*petJ*	0.05	Cytochrome c6

Copper toxicity in cells has been shown to be mediated by two key aspects: its affinity for metal binding sites in proteins, which causes protein loss of function, and ROS generation [4], [6], [12], [49], [50]. According to this, the high copper treatment induced expression of genes related to the misfolded protein stress response. Genes coding for chaperones (*groEL1*, *groEL2*, *groES*, *dnaK*, *hspA*), the signal peptidase *lepB2* and the protease *ctpB* were up-regulated (Table 2, S3 and Fig. 3B). The misfolded protein response is also induced in various stress conditions in *Synechocystis* and plays crucial roles in folding new synthesized proteins, preventing protein misfolding and/or degradation of damaged proteins [51]. Additionally, several genes related to oxidative stress were also found to be induced in our microarray data (Table 2 and S3). In *Synechoystis*, the ROS detoxification system consists of one iron containing superoxide dismutase, encoded by *sodB*, one catalase-peroxidase, encoded by *katG*, five thioredoxin-dependent peroxiredoxins and two glutathione peroxidases. The high copper treatment induced the expression of the superoxide dismutase, *sodB (slr1516)*, two peroxiredoxins, PrxII (*sll1621*) and 2-Cys-prx (*sll0755*), thioredoxin Q (*trxQ*, *slr0233*), one glutathione peroxidase, (*gpx1; slr1171*) and the NADP-thioredoxin reductase, (*ntr, slr0600*; Table 2). Furthermore, although only three genes of the PerR regulon, *aphC, (prxII; sll1621), htrA* and *perR*, were significantly up-regulated under our restrictive statistical analysis virtually all of the PerR regulon genes were induced ([39]; Tables 2, S3 and S6). To further investigate this, we analyzed ROS levels after the different copper treatments using the H_2_DCFDA dye, a fluorescent probe that reacts with several ROS including H_2_O_2_. The high copper treatment led to a ROS accumulation that was almost five times (4.7±0.6) higher than the low copper or control treatments (Fig. 3C) and similar to methyl viologen (5 µM) treated cells (3.1±0.3) [52]. All these data suggest that oxidative stress and protein damage likely mediated by copper generated ROS, are important features of the copper stress response in *Synechocystis*, as has been reported for other bacteria [53], [54], [55], [56].

**Table 2:** Selected genes induced in *Synechocystis* sp. PCC 6803 after the high copper treatment.

Locus	Gene	Ratio (3 µM/-Cu)	Description
***Photosynthetic***			
*sll0199*	*petE*	63.64	Plastocyanin
***Misfolded protein response***			
*sll0416*	*groEL2*	6.36	60 kDa chaperonin 2
*slr2075*	*groES*	5.38	10 kDa chaperonin
*slr1377*	*lepB2*	4.62	Probable signal peptidase I-2
*sll1514*	*hspA*	4.04	Small heat shock protein
*sll0170*	*dnaK*	2.95	Chaperone protein dnaK2
*slr2076*	*groEL1*	2.90	60 kDa chaperonin 1
*slr0257*	*ctpB*	5.15	Carboxyl-terminal protease
*slr0918*	*pepM*	3.45	Putative methionine aminopeptidase A (Peptidase M)
*sll1427*	*hhoB*	3.27	Protease HhoB
*slr0542*	*clpP1*	3.01	ATP-dependent Clp protease proteolytic subunit 1
*slr1204*	*htrA*	3.21	Serine protease; HtrA
***Oxidative stress response***			
*slr1738*	*perR*	10.38	Transcription regulator
*sll1621*	*ahpC*	7.79	Peroxiredoxin PrxII
*slr1171*	*gpx1*	6.41	Glutathione peroxidase
*sll0755*	*tpx*	6.06	2 Cys peroxiredoxin
*sll1980*	*txlA*	2.94	Thiol:disulfide interchange protein txlA homolog
*slr0600*	*ntr*	3.13	NADP-thioredoxin reductase
*slr0233*	*trxQ*	3.10	Thioredoxin Q
*slr1516*	*sodB*	3.23	Superoxide dismutase
***Copper resistance system***			
*sll0788*	*copM*	520.72	Putative periplasmic protein CopM
*sll0789*	*copR*	1012.24	Two-component response regulator CopR
*sll0790*	*copS*	37.08	Two-component Cu sensor histidine kinase CopS
***Heavy metal homeostasis***			
*slr0944*	*arsB*	539.56	Arsenite transport protein
*slr0945*	*arsH*	206.75	Flavoprotein
*slr0946*	*arsC*	162.58	Arsenate reductase
*slr0796*	*nrsD*	25.86	Nickel permease protein
*sll0792*	*ziaR*	9.55	Zinc transcriptional repressor
*slr0798*	*ziaA*	9.76	Zinc-transporting ATPase
*slr0797*	*corT*	7.10	Cobalt-transporting ATPase
*sll0794*	*corR*	4.08	Cobalt transcriptional repressor
*sll1920*	*pacS*	3.05	Copper-transporting ATPase
***Transport and binding proteins***			
*slr0251*	*ycf85*	6.77	ATP-binding protein of ABC transporter
*slr0544*		4.65	ATP-binding protein of ABC transporter
*sll1482*		4.31	ABC transporter permease protein
*slr0324*	*appC*	3.77	Probable ABC transporter permease protein
*slr0678*		3.57	Biopolymer transport ExbD like protein
*slr2107*	*rfbA*	2.90	Polysialic acid transport protein; KpsM.
*slr2077*		2.88	Probable ABC transporter, periplasmic binding protein
***Fe-S cluster response***			
*slr0077*	*sufS*	14.92	Probable cysteine desulfurase
*slr0075*	*sufC*	13.11	ABC transporter ATP-binding protein
*slr0076*	*sufD*	11.16	
*slr0074*	*sufB*	9.28	ABC transporter unit
*sll0088*	*sufR*	8.41	
*slr1419*	*sufE*	3.98	
*slr1846*	*grxC*	3.25	Glutaredoxin C
*sll1112*	*aroQ*	4.82	3-dehydroquinate dehydratase
*sll1470*	*leuC*	3.50	3-isopropylmalate dehydratase
*slr0958*	*cysS*	2.98	Cysteinyl-tRNA synthetase

Our transcriptional analysis also suggested changes in the integrity and permeability of membranes as a consequence of copper shock because 29 genes encoding transport function across membranes changed their expression patterns (Table 1 and 2). In addition, GSEA analysis showed a significant down regulation of gene lists related to lipoproteins synthesis, membrane biogenesis, glycoproteins, polysaccharides and porins (Fig. 3B; Table 1 and S4). Down-regulation of porins expression in response to copper stress has been previously reported in *E. coli* and two strains of marine *Synechococcus* [43], [57]. In this regard, copper uptake is thought to be a porin-mediated process, since *E. coli*, *Mycobacterium tuberculosis* and *Mycobacterium smegmatis* mutants lacking porins are more resistant to copper [57], [58], and are affected in copper acquisition at limiting concentrations [58]. The altered expression pattern of a significant number of genes related to membranes processes points to a change in membrane permeability as one of the direct effects of copper in *Synechocystis* as it has been shown in other bacteria [43], [54]. This could be a consequence of membrane damage mediated by lipid peroxidation generated by copper, as has been recently suggested for *E. coli* [59].

Lastly, the high copper treatment also led to a strong induction of the copper resistance genes. *copM* and *copR* were the top induced genes after this treatment (520 and 1012 fold, respectively), which is ten times higher induction when compared to the low copper treatment. *copS* was also highly induced when compared to the low copper treatment, indicating that the *cop* resistance system has a great range of transcriptional response to copper depending on its concentration (Table 2, S1 and S3). Additionally, this treatment also induced the import system; although *pacS* was the only gene that passed our stringent cut off, both *ctaA* (1.88-fold) and *atx1* (1.41-fold) were also induced (Tables 2 and S3). Furthermore, *petE* was induced at higher levels with respect to low copper treatment and *petJ* was the most repressed gene after the high copper treatment (Fig. 3A, Tables 1 and S3). The high level of *petE* expression together with the induction of genes related to copper import in a context of photosynthesis down-regulation suggests that plastocyanin accumulation could act as a Cu chelator under Cu overload in the thylakoid lumen, as it has been proposed in *Arabidopsis* [60]. In addition, to alleviate the increasing cytosolic copper levels, plastocyanin accumulation could also function as a copper reservoir to be later used when excess of copper stress is alleviated. Induction of the copper transporters (*pacS* and *ctaA*) and the copper chaperone (*atx1*) could be related to this increase in *petE* expression as these genes are needed to produce copper loaded plastocyanin [8], [24], [25].

### Fe-S clusters are one of the main targets of copper toxicity

Inspection of the microarray data also revealed that genes involved in Fe-S cluster biogenesis including the *suf* system (*sufS, sufC, sufD, sufB, sufE*), the regulator *sufR* and the monothiolic glutaredoxin *grxC*, were up-regulated under conditions of copper excess (Tables 2 and S3). The *suf* system is proposed to assume a supporting role in the regulation and/or assembly of Fe/S cluster in bacteria in response to oxidative stress [61] and iron starvation [62]. In higher plant chloroplast and cyanobacteria, it has been reported that the *suf* system is the main system involved in the biogenesis of the Fe/S clusters for PSI [63], [64], [65]. In cyanobacteria, the *sufR* gene is located directly upstream of the conserved *sufBCDS* operon in most sequenced cyanobacterial genomes (including *Synechocystis*) and it functions as a negative regulator of *suf* regulon in response to redox and iron stress [63]. The only *suf* gene that did not passed our stringent cut off was *sufA*, which encodes a protein that has been proposed to play a regulatory role in sensing oxidative stress in the biogenesis of iron-sulfur cluster [66], although it was also upregulated (1.75 fold induction). Furthermore, *grxC*, which encodes a monothiolic glutaredoxin containing a Fe-S cluster [67] and has been proposed to play essential roles in Fe-S repair and/or biogenesis [68], [69], was also induced. Similar findings were reported in *Bacillus subtilis*, in which microarray data for copper stress revealed a broad effect on the expression of iron-sulphur cluster biogenesis (*suf*) genes and associated pathways, such as cysteine biosynthesis and Fe-S cluster containing proteins [7]. In *E. coli*, copper toxicity produces a direct inactivation of the Fe-S clusters of the dehydratase enzymes, leading to a defect in amino acid biosynthetic pathways [6]. According to this, our transcriptional profile after the high copper treatment also exhibited the up-regulation of two genes *aroQ* and *leuC* that code for dehydratases involved in amino acids biosynthesis, as well as the cysteinyl-tRNA synthetase, *cysS* that participate in cysteine metabolism (Table 2 and S3). All these data suggest that the Fe/S cluster biogenesis and/or repair were affected by copper in *Synechocystis*.

**Figure 4:**
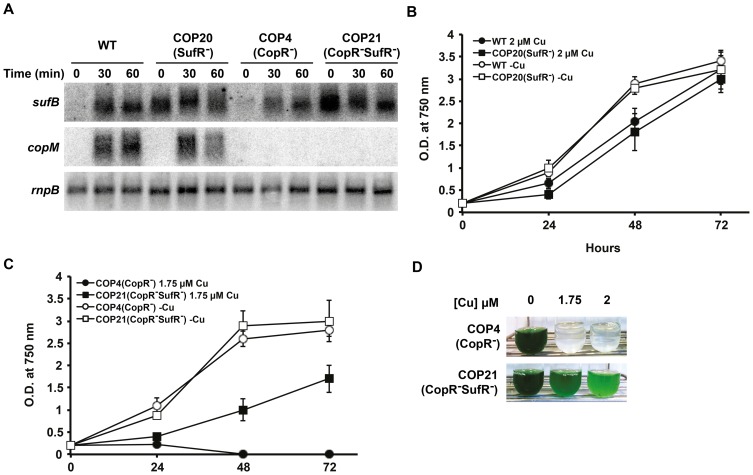
Constitutive expression of *suf* genes in COP4 (CopR^−^) increases its copper tolerance. A. Northern blot analysis of *sufB* and *copM* in WT, COP4 (CopR^−^), COP20 (SufR^−^) and COP21 (CopR^−^SufR^−^) strains. Total RNA was isolated from WT, COP4, COP20 and COP21 cells grown in BG11C-Cu medium after addition of 1 µM of copper. Samples were taken at the indicated times. The filter was subsequently hybridized with *sufB, copM* and *rnpB* (as a loading control) probes. B. Growth of WT and COP20 (SufR^−^) strains in the presence of copper. Exponentially growing cells of WT and COP20 were diluted to OD_750_ nm of 0.2 in BG11-Cu containing 2 µM of copper or without copper added. Growth was monitored following increase in OD_750 nm_ for 3 days. C. Growth of COP4 (CopR^−^) and COP21 (CopR^−^SufR^−^) strains in the presence of copper. Exponentially growing cells of COP4 and COP21 were diluted to OD_750_ nm of 0.2 in BG11-Cu containing 1.5 µM of copper or without copper added. Growth was monitored following increase in OD_750 nm_ for 3 days. D. Growth of COP4 (SufR^−^) and COP21 (CopR^−^SufR^−^) strains in the presence of copper. Exponentially growing cells of COP4 and COP21 were diluted to OD_750_ nm of 0.2 and cultured in BG11-Cu medium supplemented with the indicated copper concentration. Cultures were photographed after 3 days of growth.

In order to test the impact of *suf* system in copper resistance, we generated mutants in the *sufR* gene (*sll0088*) in WT and COP4 (CopR^−^) backgrounds, generating COP20 (SufR^−^) and COP21 (CopR^−^SufR^−^) strains respectively (Fig. S3 and S4). First of all, the two strains lacking SufR (COP20 and COP21) showed a greener colour than their parental strains (WT and COP4; Fig. S5). In fact, quantification of solvent extracted pigments from exponentially growing cultures confirmed an increase in chlorophyll levels in both strains lacking *sufR* (5.3±0.3 and 5.4±0.2 µg chl OD_750 nm_^−1^ for COP20 (SufR^−^) and COP21 (CopR^−^SufR^−^) respectively) when compared to WT (4.7±0.2 µg chl OD_750 nm_^−1^) or COP4 (CopR*^−^*; 4.8±0.3 µg chl OD_750 nm_^−1^) strains. This is in agreement with the previously published data as the *sufR* gene was originally identified as a suppressor of a single point mutant in the *psaC* gene with reduced chlorophyll content [70]. PSI reaction centers contain approximately 80% of all *Synechocystis* chlorophyll and contain several Fe-S clusters [71], and our results were consistent with the proposed role of *sufR* in regulating the biogenesis of PSI through the *suf* genes [63]. Furthermore, to validate our microarray data, the expression of *sufBCDS* operon in response to copper addition was analyzed by northern blot. Expression of the first gene in the operon, *sufB*, was analyzed in the WT, COP4 (CopR^−^), COP20 (SufR^−^) and COP21 (CopR^−^SufR^−^) strains after addition of 3 µM copper for 1 hour (Fig. 4A). *sufB* was induced in response to copper in WT and COP4 (CopR^−^) strains, while in the COP20 (SufR^−^) and COP21 (CopR^−^SufR^−^) strains the *sufB* gene was already up-regulated in untreated cultures and remained at the same levels after copper addition (Fig. 4A), in agreement with the absence of the transcriptional repressor, SufR, in these strains.

To explore whether the constitutively expression of the *sufBCDS* operon in the COP20 (SufR^−^) and COP21 (CopR^−^SufR^−^) strains was able to confer copper resistance, we tested the sensitivity of WT, COP4 (CopR^−^), COP20 (SufR^−^) and COP21 (CopR^−^SufR^−^) strains to different copper concentrations. While the COP20 (SufR^−^) strain showed a similar copper tolerance to the WT strain (Fig. 4B), the COP21 (CopR^−^SufR^−^) strain showed better growth compared to the COP4 (CopR^−^) strain, being able to grow at concentrations up to 1.75 µM of copper (Fig. 4C and 4D). These data indicates that constitutive expression of *sufBCDS* genes in the COP21 (CopR^−^SufR^−^) strain partially alleviates copper toxicity, and that Fe-S biogenesis and/or repair is an essential element for copper resistance in *Synechocystis*. This observation is further supported by the results that showed that a double mutant *atx1gshB* in this cyanobacterium, which lacks the copper metallochaperone, Atx1, and glutathione synthetase, GshB, was highly sensitive to copper and this sensitivity could be alleviated by supplementation of branched amino acids [8]. Branched amino-acids biosynthesis requires the participation of several Fe-S cluster-containing enzymes and has been proposed to be the primary target for copper toxicity in several microorganisms [6], [7]. However, the COP20 (SufR^−^) strain was not more resistant to copper than the WT strain, suggesting that the overexpression of the *sufBCDS* operon only confers a selective advantage in the absence of a copper resistance mechanism and/or that in the WT strain there is less damage to Fe-S clusters than in the COP4 (CopR^−^) strain.

### InrS controls *nrsD* transcription in response to both copper and nickel

One of the membrane related group of genes that changed its expression in response to high copper treatment were genes coding for heavy metals resistance systems. These included the arsenic resistance system (encoded by *arsBHC* operon [72]), the cobalt resistance genes *corR* (*coaR*) and *corT* (*coaA*) [73], [74]), the zinc resistance system (*ziaA* and *ziaR* [75]) and *nrsD*, the last gene of the nickel resistance operon ([73], [76]; Fig. 3D and Tables 2 and S3). All these induced genes have in common their regulation by transcriptional factors that respond to metals in the cytosol. These data suggest that under this condition (3 µM) copper accumulates in the cytosol in *Synechocystis*, at least transiently. Due to its higher affinity for proteins, copper could bind to other heavy metal transcription factors in a non-specific manner, activating them and, sub-sequentially, the genes under their control. This global regulation of metal homeostasis genes in response to copper shock has been also reported in other bacteria [53], [54], and it may allow cells to export copper by both specific and non-specific heavy metal transporters.

**Figure 5:**
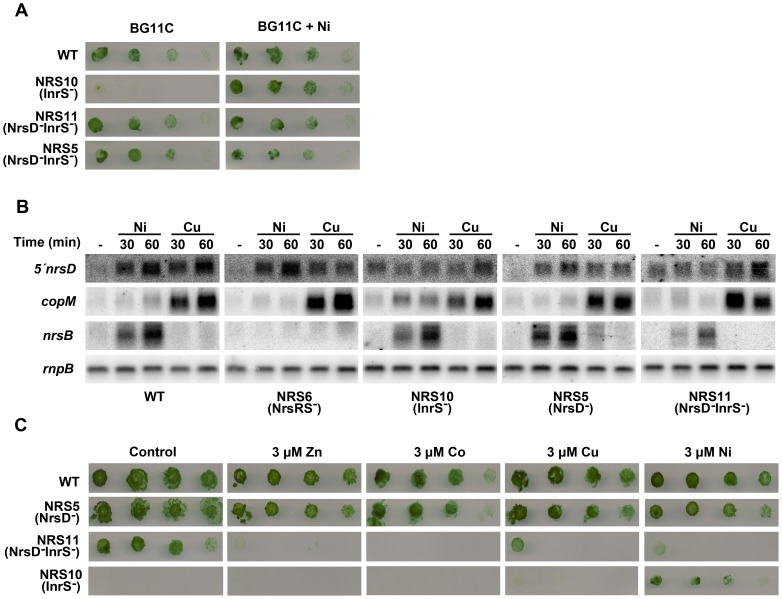
InrS is implicated in heavy metal homeostasis. A. Phenotypic characterization of mutant strains affected in *inrS* and *nrsD* genes. Growth in presence and absence of nickel was observed in WT, NRS10 (InrS^−^) and NRS11 (NrsD^−^InrS^−^) strains. Ten fold dilutions of a 1 µg chlorophyll mL^−1^ cell suspension were spotted onto both BG11C and BG11C supplemented with 1 µM of nickel. Plates were photographed after 5 d of growth. B. Northern blot analysis of the expression of *nrsD* in WT, NRS5 (NrsD^−^), NRS6 (NrsRS^−^), NRS10 (InrS^−^) and NRS11 (NrsD^−^InrS^−^) strains. Total RNA was isolated from WT, NRS5, NRS6, NRS10 and NRS11 strains grown in BG11C-Cu medium and exposed for 60 min to 3 µM of indicated metals ions. Control cells were not exposed to added metals (-). The filter was hybridized with *5′nrsD*, *copM*, *nrsB* and *rnpB* (as a loading control) probes. C. Phenotypic characterization of WT, NRS5 (NrsD^−^), NRS10 (InrS^−^) and NRS11 (NrsD^−^InrS^−^) mutant strains. Tolerance of WT, NRS5, NRS10 and NRS11 strains to different metals was examined. Ten fold dilutions of a 1 µg chlorophyll mL^−1^ cell suspension cell were spotted onto BG11C, supplemented with the indicated metals ions concentrations. Plates were photographed after 5 d of growth.

The only gene mentioned above that is not exclusively controlled by cytosolic regulators is *nrsD*, which encodes for a nickel permease belonging to the major facilitator superfamily of transport proteins, and is part of the *nrsBACD* operon. This operon is involved in nickel resistance in *Synechocystis* and is controlled by the NrsRS two-component system [73], [77]. Although *nrsD* was induced by copper neither *nrsBAC* nor *nrsRS* genes were induced after this treatment (Fig. 3D). These data were confirmed by northern blot analysis of *nrsD* (using a probe for *nrsD, nrsD5′*, that comprises 310 bp before the insertion point for the *CK*.1 cassette used to construct the NRS5 and NRS11 strains, see below and Fig. S6) and *nrsB* genes. The *nrsD* transcript was induced in both copper and nickel treatments while *nrsB* was only induced by nickel addition (Fig. 5B). Recently, it has been described that *nrsD* has its own promoter and is also regulated by the cytosolic nickel sensing transcription factor InrS, which belongs to the CsoR family of transcription factors [76]. Even more, *nrsD* was still induced after copper addition in the NRS6 (NrsRS^−^) mutant strain (which lacks the NrsRS system) while *nrsB* was not induced (Fig. 5B). This suggests that *nrsD* is under the control of another system in response to copper. The most likely regulator involved in the induction of *nrsD* gene under this condition is the InrS repressor. This family of transcription factors were initially identified as copper responsive [19], [78], [79], and InrS contains the conserved residues to bind Cu. In fact, InrS binds Cu(I) more tightly than Ni(II), although it was proposed that this protein does not have access to Cu *in vivo* under steady-state conditions, because all Cu is buffered in *Synechocystis* cytoplasm [76], but responds to both Cu and Zn after a short challenge [80], therefore corroborating our results (Fig. 5B). In order to study the role of InrS in copper homeostasis, mutant strains with an interrupted *inrS* gene were constructed in both WT (generating the NRS10 strain) and NRS5 (NrsD^−^; generating the NRS11 strain) backgrounds. The NRS10 (InrS^−^) strain presented a slow growth phenotype, which was partially alleviated by Ni addition to the media and expressed *nrsD* constitutively (Fig. 5A and B; [76]). This suggests that the slow growth phenotype observed in NRS10 (InrS^−^) strain could be consequence of its low Ni content (our unpublished results and [76]), probably due to the constitutive expression of *nrsD*. In agreement with this, the NRS11 (InrS^−^NrsD^−^) strain grew as the WT in BG11C and also expressed *nrsD* constitutively (Fig. 5A and B; the expression was detected because the probe covers the sequence before the insertion point of CK1). In order to study whether InrS has a role in other metals metabolism, the growth of WT, NRS5 (NrsD^−^), NRS10 (InrS^−^) and NRS11 (InrS^−^NrsD^−^) strains was analyzed in the presence of different metals. The NRS11 (InrS^−^NrsD^−^) was extremely sensitive to the presence of all metals tested, unlike the NRS5 (NrsD^−^) and WT strains (Fig. 5C) that were not affected at this metal concentrations, while the NRS10 (InrS^−^) strain was only able to grow in presence of nickel. These findings suggest that InrS has a central role in metal homeostasis, including copper, in *Synechocystis* probably controlling other elements beyond *nrsD* that will require further studies.

### CopRS only controls *copMRS* and *copBAC* expression in response to copper

**Figure 6:**
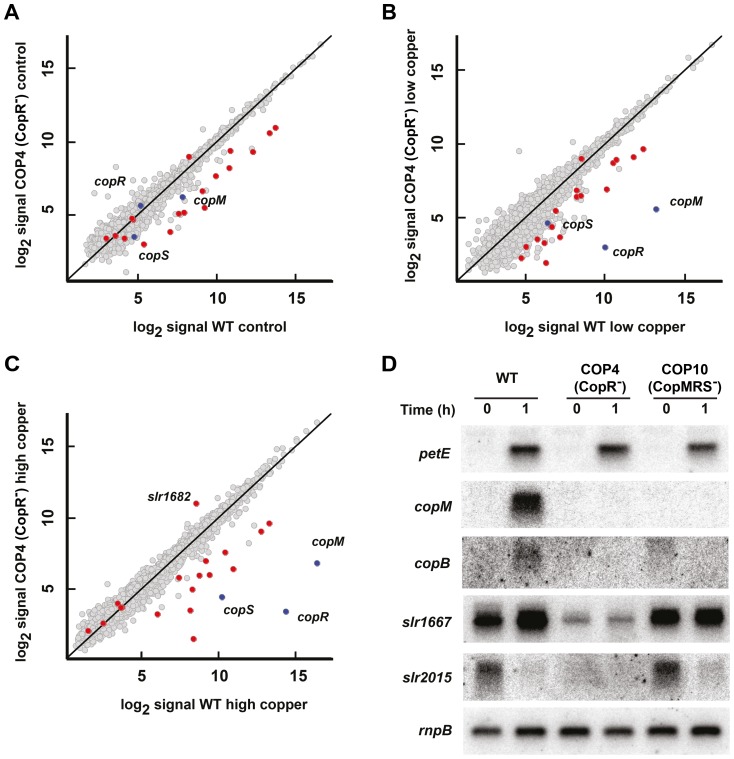
Global responses to copper in the COP4 (CopR^−^) mutant strain. A. Scatter plot showing comparison between expression profiles of COP4 (CopR^−^; y axis) and WT (x axis) in untreated samples. Data represents the average signal of two hybridizations for COP4 and four hybridizations for WT. In red are colored genes that are statistically regulated in COP4 strain in all treatments. In blue is colored the *copMRS* operon. B. Scatter plot showing comparison between expression profiles of COP4 (CopR^−^; y axis) and WT (x axis) treated with 0.3 µM copper for 1 h. Data represents the average signal of two hybridizations for COP4 and WT. Colours were used as in A. C. Scatter plot showing comparison between expression profiles of COP4 (CopR^−^; y axis) and WT (x axis) treated with 3 µM copper for 1 h. Data represents the average signal of two hybridizations for COP4 and WT. Colours were used as in A. D. Northern blot analysis of *copM*, *slr1667*, *slr2015* and *petE* in WT, COP4 (CopR^−^) and COP10 (CopMRS^−^) strains. Total RNA was isolated from WT, COP4 and COP10 cells grown in BG11C-Cu medium and exposed to 3 µM of copper for 1 h. The filter was hybridized with *copM*, *slr1667*, *slr2015*, *petE* and *rnpB* (as a loading control) probes.

Finally we have also analyzed the global gene expression profile in the COP4 (CopR^−^) strain, a mutant strain that lacks the CopRS two-component system, which is essential for copper resistance and regulation of *copMRS* and *copBAC* operons [21]. The COP4 (CopR^−^) strain carries a deletion of the *copM_1_R_1_S_1_* and an insertion in the *copR_2_* gene and it was previously characterized to be copper sensitive, while mutants lacking *copM_1_R_1_S_1_* or *copM_2_R_2_S_2_* were indistinguishable from the WT strain both in copper resistance [21] and *copM* expression (Fig. S7). The statistical analysis showed that only 20 *ORFs* were significantly down-regulated in any of the conditions assayed in the COP4 (CopR^−^) strain. The only exception was a putative transposase encoded by *slr1682*, which was up-regulated after the high copper treatment (Fig. 6, Table S5). Most of these genes corresponded to 4 operons, that with the exception of *copMRS*, were already down-regulated in control conditions, suggesting that their expression was not copper dependent (Fig. 6A). To verify this hypothesis, and the microarray results, the expression of the first gene in the operons *slr1667* (*slr1667-slr1668*), *slr2015* (*slr2015-slr2016-slr2017-slr2018*) and *copM* (both *copM_1_R_1_S_1_ and copM_2_R_2_S_2_*) was analyzed in response to copper in WT, COP4 (CopR^−^) and COP10 (CopMRS^−^) strains (a strain which carries a deletion of both *copM_1_R_1_S_1_* and *copM_2_R_2_S_2_* and shows the same copper sensitivity phenotype to COP4 (CopR^−^) strain; Fig. S3 and S8) by northern blot. This showed that *petE* was induced in all strains and both *copM* (*copM_1_* (*sll0788*) and *copM_2_* (*slr6039*)) and *copB (slr6038)* were only expressed in the WT strain (Fig. 6D), as expected. In contrast, both *slr1667* and *slr2015* have the same expression pattern in both WT and COP10 (CopMRS^−^) strains and were expressed at lower level in the COP4 (CopR^−^) strain in untreated conditions. These two genes were down-regulated to similar levels to those of the COP4 (CopR^−^) strain in WT and COP10 (CopMRS^−^) strains after copper addition (Fig. 6D). These genes together with *slr0442*, *ssr2787* and *ssr2848* have been also described to be repressed in mutants lacking either the cyanobacterial cAMP receptor proteins, SynCRP1 [81], [82], or the cyanobacterial homologue of the RNA chaperone Hfq [83]. These suggested that the COP4 strain could carry a secondary mutation in one of these two genes. However, sequencing of these genes did not show any differences between WT and COP4 (CopR^−^) strains, pointing to an additional mutation affecting expression of these genes in COP4 (CopR^−^) but not to copper related genes. All these data suggest that CopRS only controls *copMRS* and *copBAC* operons both under our standard conditions and after 1 h of copper stress, although we can not exclude the possibility that this system could control other *Synechocystis* plasmids genes as *copBAC*, since our microarray did not contain probes for genes located in these plasmids. These results were further supported by a bioinformatics search for the putative CopR DNA binding sequences (TTCATN_4–5_TTCAT; [21]) in the *Synechocystis* genome that were only found, in addition to the *cop* promoters, upstream the *mntC* gene and in the divergent promoters located between *nrsRS* and *nrsBACD* operons [77]. However, none of these genes showed any differential expression patterns in any of the strains used (Tables S1 and S3, Figures 3D and 5B), suggesting that there should be more elements involved in the regulation of these promoters.

These results contrast with the previously data showing that the Hik31-Rre34 two component system (designated CopRS here) is involved in the responses to glucose both under continuous light or under light/dark cycles, with different roles for the plasmid and genomic copies of these genes. Our mutant strains did not show any defects after glucose addition and mutants lacking only one of the copies of these genes were phenotypically identical to the WT strain [21] and no change in pigmentation was observed in any of our strains (Fig. 4; and our unpublished observations). These differences could be attributable to different strain backgrounds, as glucose sensitivity has been shown to be variable between different WT strains [26], [84], [85], and/or media formulations. In fact, It has also been reported that *copMRS* were induced by conditions that altered the redox state of the cell [40], [41], [86], but we have shown that at least after DBMIB addition and nitrogen starvation it only happens in copper containing media [21], [35], which suggests that most of the functions related to these genes are related to copper. Therefore some of the phenotypes attributed to be controlled by the CopRS (Hik31-Rre34) could be a consequence of the copper released from degradation of oxidized plastocyanin [35], as many of these conditions will alter photosynthetic electron transport and probably lead to the accumulation of oxidized plastocyanin.

## Conclusions

**Figure 7:**
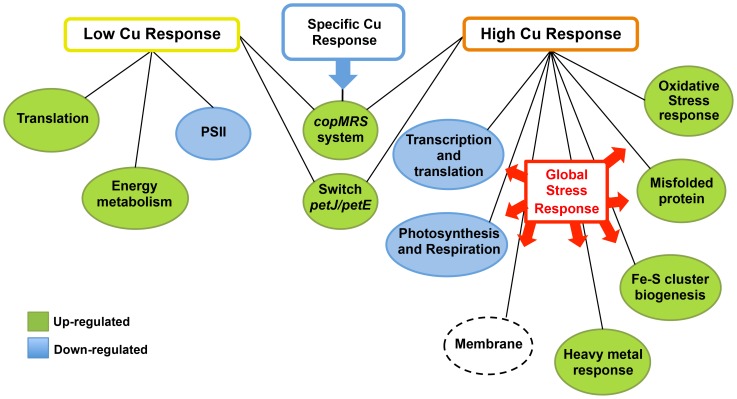
A Schematic representation depicting gene sets transcriptionally regulated by copper in *Synechocystis*. Group of up-regulated genes are shown in green and group of down-regulated genes are shown in blue. Dashed line represents a group that contains both up- and down-regulated genes.

In this work we have reported the transcriptional profiles of the WT and a *copR* mutant (COP4) *Synechocystis* strains in response to low and high copper concentration treatments. The low copper treatment (0.3 µM) revealed a slight induction of cell anabolism, mainly cyclic photosynthesis, through up-regulation of genes related to energy metabolism and translation and repression of PSII genes (Fig. 7). On the other hand, the toxic copper concentration catalyzed the formation of ROS and led to a general stress response, which included the repression of genes related to photosynthesis, respiration and growth, and the induction of chaperones and oxidative stress related genes. This treatment also affected expression of a high number of genes related to biogenesis and transport across the membrane, heavy metal resistance and Fe-S cluster biogenesis and/or repair indicating that copper markedly affected these processes. Additionally, both copper treatments (0.3 and 3 µM) had in common the *petJ*/*petE* transcriptional switch and the induction of *copMRS* operon, which we have defined as the specific copper response in *Synechocystis* (Fig. 7). Furthermore, the induction of Fe-S cluster repair/biosynthesis genes has an important role in copper toxicity in *Synechocystis*, since a double mutant strain lacking both *copR* and *sufR* (the COP21 strain) that expressed constitutively the *sufBCDS* operon, was more resistant to copper than the *copR* mutant strain (COP4). Moreover, we have also shown that InrS, a CsoR transcriptional factor, controls *nrsD* expression not only in response to nickel but also to copper. In addition, we have shown that InrS has an important role in heavy metals homeostasis, including copper, in *Synechocystis*. Finally, the analysis of the COP4 strain (CopR^−^) revealed that *copMRS* and *copBAC* operons are the only targets of the CopRS two-component system in response to copper.

## Materials and Methods

### Strains and culture conditions

*Synechocystis* strains used in this work are listed in Table 3. All *Synechocystis* strains used in this work were grown photoautotrophically on BG11C-Cu (lacking CuSO_4_) medium [31] at 30°C under continuous illumination (50 µE m^−2^ s^−1^) and bubbled with a stream of 1% (v/v) CO_2_ in air. For plate cultures, media was supplemented with 1% (wt/vol) agar. Kanamycin, chloramphenicol and spectinomycin were added to a final concentration of 50 µg mL^−1^, 20 µg mL^−1^ and 5 µg mL^−1^, respectively. Experiments were performed using cultures from the mid-logarithmic phase (3–4 µg chlorophyll mL^−1^) in BG11C-Cu medium supplemented with indicated amounts of CuSO_4_, NiSO_4_, CoCl_2_, ZnCl_2_ and Methyl viologen (MV) when required.

**Table 3:** *Synechocystis* strains used in this study.

STRAIN	PREVIOUS NAME	RELEVANT PHENOTYPE	GENOTYPE	MUTATED ORFS	SOURCE
**WT**	*-*	*-*	*Synechocystis* sp. PCC 6803	-	Lab collection From Institute Pasteur.
**COP1**	GCOP	*-*	*ΔcopM_1_R_1_S_1_::SpΩ*	*sll0788, sll0789, sll0790*	[21]
**COP4**	COPR	CopR*^−^*	*ΔcopM_1_R_1_S_1_::SpΩ copR_2_::C.C1*	*slr6040, sll0788, sll0789, sll0790*	[21]
**COP5**	PCOP	*-*	*ΔcopM_2_R_2_S_2_::C.K1*	*slr6039, slr6040, slr6041*	[21]
**COP10**	*-*	CopMRS*^−^*	*ΔcopM_2_R_2_S_2_::C.K1 ΔcopM_1_R_1_S_1_::C.C1*	*slr6039, slr6040, slr6041, sll0788, sll0789, sll0790*	This study
**COP20**	*-*	SufR*^−^*	*sufR::C.K1*	*sll0088*	This study
**COP21**	*-*	SufR*^−^* CopR*^−^*	*ΔcopM_1_R_1_S_1_::SpΩ copR_2_::C.C1 sufR::C.K1*	*slr6040, sll0788, sll0789, sll0790, sll0088*	This study
**NRS5**	*nrsD::CK1+*	NrsD*^−^*	*nrsD::C.K1*	*slr0796*	[73]
**NRS6**	*nrsRS::CK1+*	NrsRS*^−^*	*ΔnrsRS:: C.K1*	*sll0797, sll0798*	[77]
**NRS10**	*-*	InrS*^−^*	*ΔinrS::SpΩ*	*sll0176*	This study
**NRS11**	*-*	InrS*^−^*NrsD*^−^*	*nrsD::C.K1 ΔinrS::SpΩ*	*slr0796, sll0176*	This study

*E. coli* DH5α cells were grown in Luria broth medium and supplemented with 100 µg ml^−1^ ampicillin, 50 µg ml^−1^ kanamycin, 20 µg ml^−1^ chloramphenicol and 100 µg ml^−1^ spectinomycin when required.

### Insertional mutagenesis of *Synechocystis* genes

For the *sll0088, sufR*, insertional mutant, an 1135-bp DNA band amplified with oligonucleotides SUFRF and SUFRR was cloned into pGEMT to generate pSUFR1. Then antibiotic resistance C.K1 cassette [87] was inserted into an *Eco*RV site, generating pSUFR2, and this plasmid was used to transform both WT and COP4 strains generating COP20 and COP21 mutant strain respectively. For the NRS10 (*inrS* mutant) and NRS11 (*inrS* and *nrsD* double mutant) mutants strains a 1211-bp DNA band, excluding the *inrS* ORF but containing the flanking regions, was amplified by overlapping PCR reactions using oligonucleotides pairs CSOR5L-CSOR5R and CSOR3L-CSOR3R and cloned into pGEMT to generate pCSOR1.1. Then a spectinomycin (SpΩ) resistance cassette was introduced into the EcoRV site generated during the overlapping PCR to generate pCSOR7 and this plasmid was transformed into WT or NRS5 (described as *nrsD*::CK1 in [73]) strains. To generate the COP10 mutant strain, a 3032-bp DNA band was amplified with oligonucleotides ΔcopM1 and ΔcopS4 and was cloned into pGEMT to generate pCOPRS9. Then an SpΩ resistance cassette was introduced between the *Sal*I-*BstE*II sites that were made blunt ended by Klenow DNA polymerase, generating pCOPRS11. Finally this plasmid was used to transform the COP1 strain [21].

All plasmids were incorporated by homologous recombination in the genome and complete segregation of the mutants generated in this work was checked by PCR using the oligonucleotides shown in Table S7.

### Minimal inhibitory concentration (MIC) determination

The MIC for Cu was calculated as the lowest concentration at which there was no growth after 24 h. *Synechocystis* was grown in tubes of 25 ml at 30°C inoculated at OD_750_ nm of 0.6, in duplicate, with different CuSO_4_ concentrations. After 24 h, the OD at 750 nm was and the MIC was determined.

### ROS determination

ROS analysis was performed following the protocol previously described [88], [89]. Total protein extracts (250–500 µg total protein) of *Synechocystis* cultures in the mid-exponential growth phase (3 to 4 µg chlorophyll mL^−1^) under different conditions were used for ROS quantification. Each measurement was performed on three equal aliquots, one of them containing 100 mM ascorbate used as background signal. Samples were incubated for 15 min at 25°C. Then, 2′,7′-dichlorodihydrofluorescein diacetate (H_2_DCFDA) (Invitrogen catalog #D399) in dimethyl sulfoxide (DMSO) was added to a final concentration of 25 µM and incubated for 30 min at 30°C. Fluorescence was measured using a Cary Eclipse fluorescence spectrophotometer (Varian) with excitation/emission wavelengths set up to 485 and 525 nm, respectively. For each experimental sample the ascorbate- background was subtracted. The obtained values were expressed as relative fluorescence units per microgram of protein. Each experiment was performed three independent times.

### RNA Isolation and Northern-blot analysis

Total RNA was isolated from 30 mL samples of *Synechocystis* cultures in the mid-exponential growth phase (3 to 4 µg chlorophyll mL^−1^). Extractions were performed by vortexing cells in presence of phenol-chloroform and acid-washed baked glass beads (0.25–0.3 mm diameter) as previously described [47]. 5 µg of total RNA was loaded per lane and electrophoresed in 1.2% agarose denaturing formaldehyde gels [90] and transferred to nylon membranes (Hybond N-Plus; GE Healthcare). Prehybridization, hybridization, and washes were in accordance with GE Healthcare instruction manuals. Probes for Northern blot hybridization were synthesized by PCR using oligonucleotide pairs: petEF-petER, petJF-petJR, copM1F-copM1R, copBF-copBR, slr2015F-slr2015R, slr1667F-slr1667R, nrsBF-nrsBR, NRP3-NRP1, sufRF-sufRR, sufBF-sufBR (see Table S7) for *petE, petJ, copM, copB, slr2015, slr1667, nrsB, 5′nrsD, sufR and sufB*, respectively. As a control, in all cases the filters were stripped and re-probed with a 580-bp *Hind*III-*Bam*HI probe from plasmid pAV1100 containing the constitutively expressed RNase P RNA gene (*rnpB*) from *Synechocystis* (Vioque, 1992). DNA probes were ^32^P labeled with a random-primer kit (Amersham Biosciences) using [α-^32^P] dCTP (3,000 Ci/mmol). Hybridization signals were quantified with a Cyclone Phosphor System (Packard). Each experiment was performed at least two independent times.

### Microarray hybridization, bioinformatics and data analysis

For microarray analysis 0.2 µg of RNA were transformed to cRNA using Low Input Quick Amp WT Labeling Kit from Agilent. cRNA was labeled with Cy3 and labeled cRNA was applied to 8×15K arrays Agilent arrays. Signal intensities for probes were obtained from the scanned microarray image using Agilent Technologies' Feature Extraction software and quantile normalized. Differentially expressed genes were selected using Limma [91] implemented in One Channel GUI with a *p*<0.01 and at least 2.5 fold change. Gene groups differentially expressed in different genotypes were identified using GSEA tool [32] using hand-compiled gene lists (Table S8) that include functional categories from cyanobase, GO annotation and literature curated gene list (see supplementary material). The data discussed in this publication have been deposited in NCBI's Gene Expression Omnibus and are accessible through GEO Series accession number GSE51671. (http://www.ncbi.nlm.nih.gov/geo/query/acc.cgi?token=ebcpyuqknbytvul&acc=GSE51671).

## Supporting Information

Figure S1 **The switch between** ***petE*** **/** ***petJ*** **genes at 0.3 µM of copper.**
(PDF)

Figure S2 ***copM*** **is expressed in cells cultured in standard BG11C medium under steady-state conditions.**
(PDF)

Figure S3 **Schematic representation of the** ***Synechocystis*** **mutants strains affected in the** ***copMRS*** **genes used in this work.**
(PDF)

Figure S4 **Schematic representation of the** ***Synechocystis*** **mutants strains affected in the** ***sufR*** **gene used in this work.**
(PDF)

Figure S5 **Changes in pigmentation in the** ***sufR*** **mutant strains.**
(PDF)

Figure S6 **Schematic representation of the** ***Synechocystis*** **mutants strains affected in the** ***nrs*** **and** ***inrS*** **genes used in this work.**
(PDF)

Figure S7 ***copM*** **expression in response to copper is not altered in COP1 and COP5 strains.**
(PDF)

Figure S8 **COP10 (CopMRS^−^) and COP4 (CopR^−^) show the same copper sensitivity phenotype.**
(PDF)

Table S1 **List of differentially expressed genes in low copper treatment in** ***Synechocystis*** **sp. PCC 6803.**
(XLSX)

Table S2 **GSEA analysis for low copper treatment.**
(XLSX)

Table S3 **List of differentially expressed genes in high the copper treatment in** ***Synechocystis*** **sp. PCC 6803.**
(XLSX)

Table S4 **GSEA analysis for high copper treatment.**
(XLSX)

Table S5 **List of differentially expressed genes in** ***Synechocystis*** **WT and the COP4 mutant strain.**
(XLSX)

Table S6 **The PerR regulon genes after the high copper treatment.**
(DOCX)

Table S7 **Oligonucleotides used in this work.**
(DOCX)

Table S8 **Hand curated genes used in the GSEA analysis.**
(XLSX)
