# Supplementary material for: Global Transcriptional Profiles of the Copper Responses in the Cyanobacterium Synechocystis sp. PCC 6803
Source: PLoS One. 2014 Sep 30;9(9):e108912. doi: 10.1371/journal.pone.0108912 (PMC4182526; doi:10.1371/journal.pone.0108912)
Supplement: Figure S1 — The switch between petE / petJ genes at 0.3 µM of copper. (PDF) [file pone.0108912.s001.pdf]

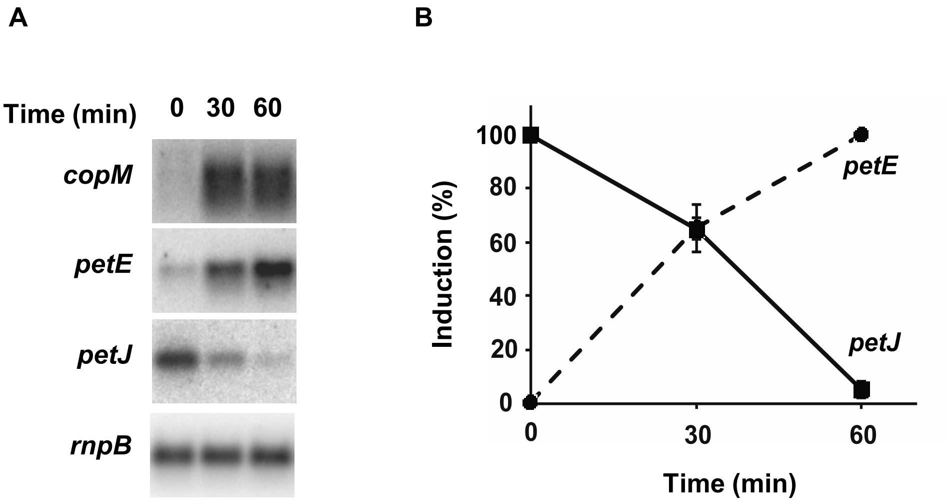


**Figure S1. The switch between *petE*/*petJ* genes at 0.3 µM of copper.**

A. Northern blot analysis of the expression of *copM*, *petE*, and *petJ* in response to non-toxic copper addition in wild-type. Total RNA was isolated from cells grown in BG11C-Cu medium after addition of copper 0.3 µM. Samples were taken at the indicated times. The filters were hybridized with *copM*, *petE*, and *petJ* probes and subsequently stripped and rehybridized with an *rnpB* probe as a control.

B. Quantification of relative mRNA levels of *petE* and *petJ* in response to 0.3 µM Cu addition. Radioactive signals of three independent experiments were quantified and averaged. RNA levels were normalized with the rnpB signal. Plots of relative mRNA levels versus time were drawn; error bars represent SD. *petE* (circles) and *petJ* gene (squares).
