# Supplementary material for: Global Transcriptional Profiles of the Copper Responses in the Cyanobacterium Synechocystis sp. PCC 6803
Source: PLoS One. 2014 Sep 30;9(9):e108912. doi: 10.1371/journal.pone.0108912 (PMC4182526; doi:10.1371/journal.pone.0108912)
Supplement: Figure S2 — copM is expressed in cells cultured in standard BG11C medium under steady-state conditions. (PDF) [file pone.0108912.s002.pdf]

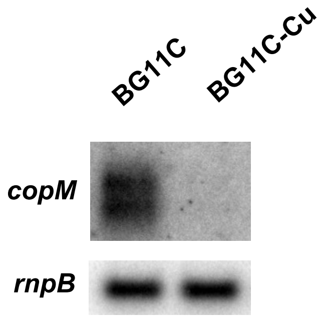

Figure S2. *copM* is expressed in cells cultured in standard BG11C medium under steady-state conditions. Northern-blot analysis of the expression of *copM* in wild-type cells cultured in either standard copper concentration medium (BG11C) or low copper medium (BG11C-Cu). Total RNA was isolated from cells after two cycles of growth up to mid-log phase in BG11C or BG11C-Cu. The filter was hybridized with *copM*, and subsequently stripped and re-hybridized with an *rnpB* probe as a control
