# Supplementary material for: Global Transcriptional Profiles of the Copper Responses in the Cyanobacterium Synechocystis sp. PCC 6803
Source: PLoS One. 2014 Sep 30;9(9):e108912. doi: 10.1371/journal.pone.0108912 (PMC4182526; doi:10.1371/journal.pone.0108912)
Supplement: Figure S3 — Schematic representation of the Synechocystis mutants strains affected in the copMRS genes used in this work. (PDF) [file pone.0108912.s003.pdf]

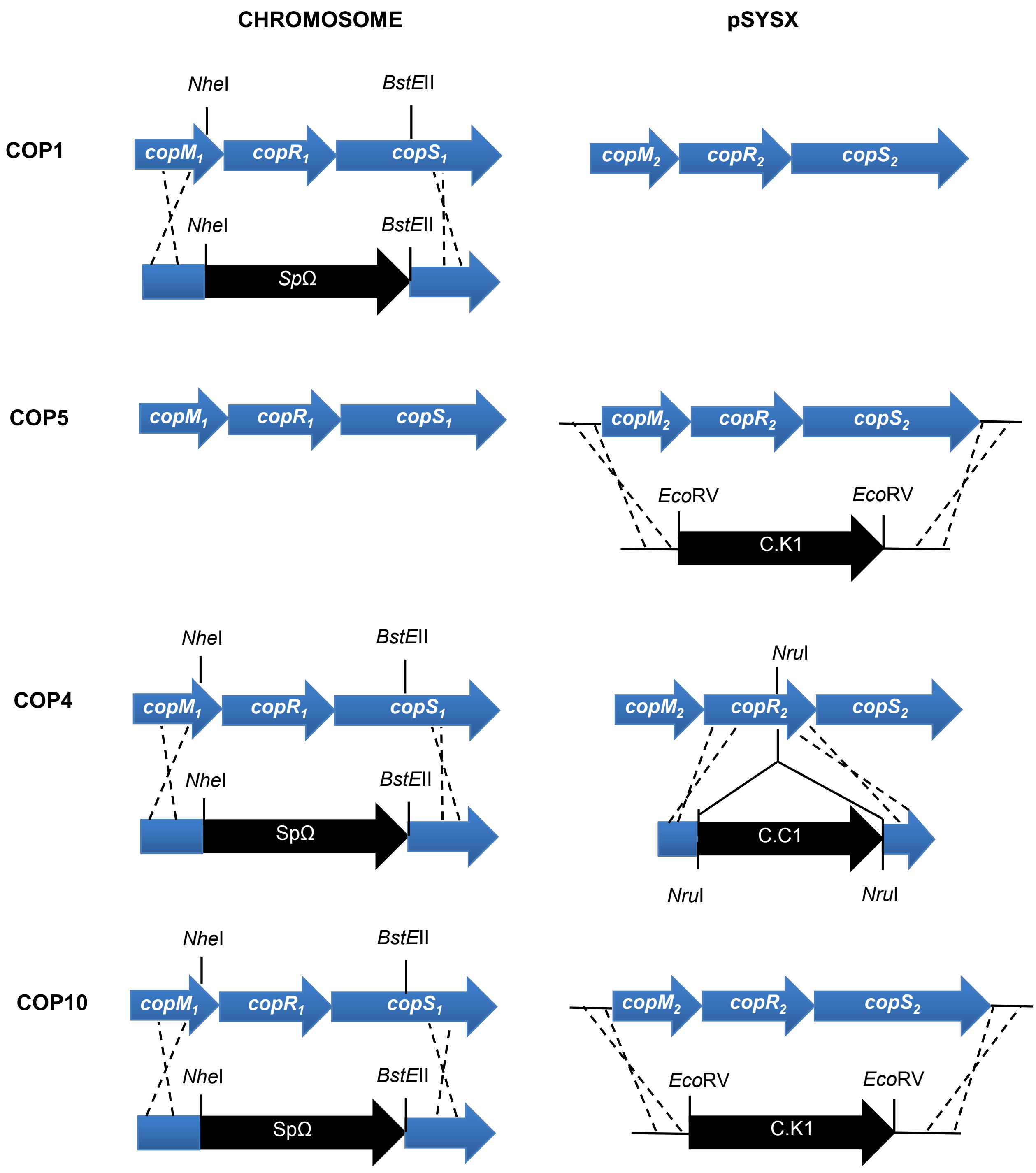


**Figure S3. Schematic representation of the *Synechocystis* mutants strains affected in the *copMRS* genes used in this work.** Schematic representation of the *cop* genes region in both genomic (*copM*$_1$*R*$_1$*S*$_1$) and pSYSX plasmid (*copM*$_2$*R*$_2$*S*$_2$) in the COP mutant strains. The *C.K1* and *Sp*Ω and *C.C1* cassettes were inserted in each mutant strain at the indicated restriction sites. COP1, COP5 and COP4 were previously published and designated as GCOP, PCOP and COPR respectively (Giner-Lamia et. al 2012 Plant Physiology 159:1806-1818). Crossed dashed lines show homolog recombination sites.
