# Supplementary material for: Global Transcriptional Profiles of the Copper Responses in the Cyanobacterium Synechocystis sp. PCC 6803
Source: PLoS One. 2014 Sep 30;9(9):e108912. doi: 10.1371/journal.pone.0108912 (PMC4182526; doi:10.1371/journal.pone.0108912)
Supplement: Figure S4 — Schematic representation of the Synechocystis mutants strains affected in the sufR gene used in this work. (PDF) [file pone.0108912.s004.pdf]

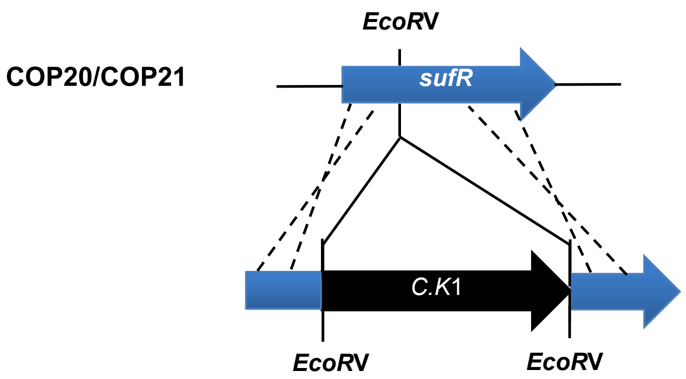

Figure S4. Schematic representation of the *Synechocystis* mutants strains affected in the *sufR* gene used in this work. Schematic representation of the *sufR* gene region in both COP20 and COP21 mutant strains. The *C.K1* cassette was inserted in the WT and the COP4 ($CopR^-$) mutant strain at the indicated restriction site to generate the COP20 and the COP21 mutant strains respectively. Crossed dashed lines show homolog recombination sites.
