# Supplementary material for: Global Transcriptional Profiles of the Copper Responses in the Cyanobacterium Synechocystis sp. PCC 6803
Source: PLoS One. 2014 Sep 30;9(9):e108912. doi: 10.1371/journal.pone.0108912 (PMC4182526; doi:10.1371/journal.pone.0108912)
Supplement: Figure S5 — Changes in pigmentation in the sufR mutant strains. (PDF) [file pone.0108912.s005.pdf]

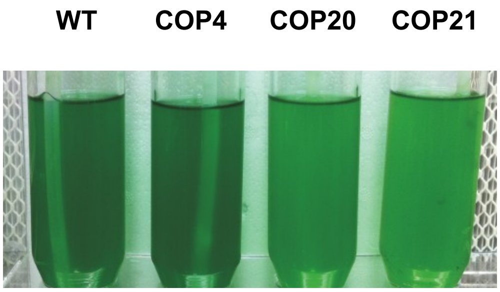

Figure S5. Changes in pigmentation in the *sufR* mutant strains.
Cells of WT, COP4 ($CopR^-$), COP20 ($SufR^-$) and COP21 ($CopR^-SufR^-$) were diluted to $OD_{750\ nm}$ of 0.2 and cultured in BG11C-Cu medium up to the exponential phase, $OD_{750\ nm}$ of 0.8. Cultures were photographed under this condition.
