# Supplementary material for: Global Transcriptional Profiles of the Copper Responses in the Cyanobacterium Synechocystis sp. PCC 6803
Source: PLoS One. 2014 Sep 30;9(9):e108912. doi: 10.1371/journal.pone.0108912 (PMC4182526; doi:10.1371/journal.pone.0108912)
Supplement: Figure S6 — Schematic representation of the Synechocystis mutants strains affected in the nrs and inrS genes used in this work. (PDF) [file pone.0108912.s006.pdf]

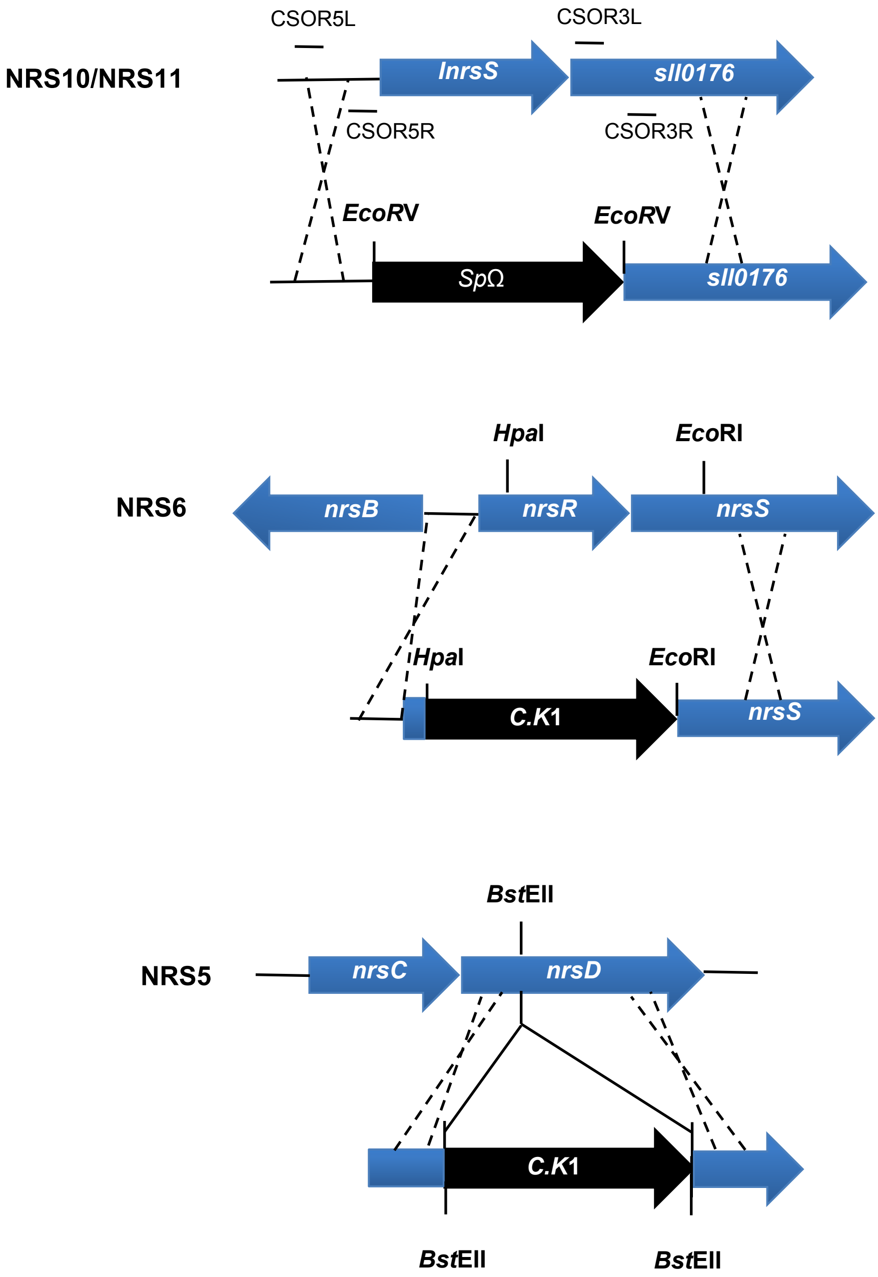

Figure S6. Schematic representation of the *Synechocystis* mutants strains affected in the *nrs* and *inrS* genes used in this work. Schematic representation of the *nrs* and *inrS* genes region in the NRS mutant strains. The *Sp*Ω and *C.K*1 cassettes were inserted at the indicated restriction site. NRS5 and NRS6 were previously published and designated as NRSD, NRSRS+, respectively (García-Domínguez et. al 2000. J Bacteriol. Mar;182:1507-1514; Lopez-Maury et. al 2002 Molecular Microbiology 43: 247-256). Crossed dashed lines show homolog recombination sites. Oligonucleotides uses in this work for generate both NRS10 and NRS11 mutant strains are shown.
