# Supplementary material for: Global Transcriptional Profiles of the Copper Responses in the Cyanobacterium Synechocystis sp. PCC 6803
Source: PLoS One. 2014 Sep 30;9(9):e108912. doi: 10.1371/journal.pone.0108912 (PMC4182526; doi:10.1371/journal.pone.0108912)
Supplement: Figure S7 — copM expression in response to copper is not altered in COP1 and COP5 strains. (PDF) [file pone.0108912.s007.pdf]

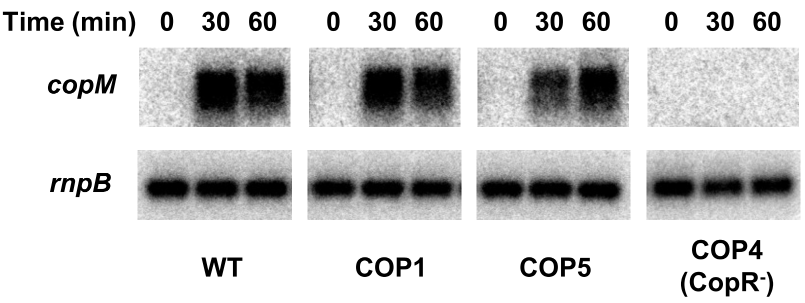

Figure S7. ***copM* expression in response to copper is not altered in COP1 and COP5 strains.** Northern blot analysis of the expression of *copM* in response to 3 μM copper in WT, COP1, COP4 ($CopR^-$) and COP5 strains. Total RNA was isolated from cells grown in BG11C-Cu medium after addition of copper 3 μM. Samples were taken at the indicated times. The filter was subsequently with *copM* and *rnpB* (as loading control) probes.
