# Supplementary material for: Global Transcriptional Profiles of the Copper Responses in the Cyanobacterium Synechocystis sp. PCC 6803
Source: PLoS One. 2014 Sep 30;9(9):e108912. doi: 10.1371/journal.pone.0108912 (PMC4182526; doi:10.1371/journal.pone.0108912)
Supplement: Figure S8 — COP10 (CopMRS−) and COP4 (CopR−) show the same copper sensitivity phenotype. (PDF) [file pone.0108912.s008.pdf]

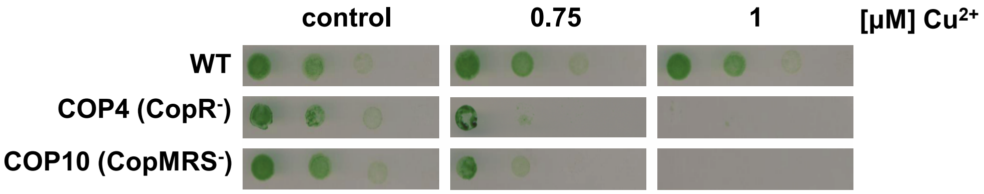

Figure S8. COP10 (CopMRS$^-$) and COP4 (copR$^-$) show the same copper sensitivity phenotype.

Growth of WT, COP4 and COP10 strains the presence or absence of copper. Ten fold dilutions of a 1 μg chlorophyll mL^{-1} cell suspension were spotted onto BG11C-Cu supplemented with 0.75 and 1 μM of copper. Plates were photographed after 5 days of growth.
