# Supplementary material for: Global Transcriptional Profiles of the Copper Responses in the Cyanobacterium Synechocystis sp. PCC 6803
Source: PLoS One. 2014 Sep 30;9(9):e108912. doi: 10.1371/journal.pone.0108912 (PMC4182526; doi:10.1371/journal.pone.0108912)
Supplement: Table S6 — The PerR regulon genes after the high copper treatment. (DOCX) [file pone.0108912.s014.docx]

Table S6. PerR regulon genes after high copper treatment.

| **Locus** | **Gene** | **Ratio** | **Description** |
| --- | --- | --- | --- |
|  |  |  |  |
| *slr1738* | *perR* | 10.38 | transcription regulator Fur family |
| *slr1739* | *psb28* | 2.10 | photosystem II 13 kDa protein homolog |
| *sll1620* |  | 1.75 | hypothetical protein |
| *sll1621* | *aphC* | 7.79 | AhpC/TSA family protein |
| *ssl2667* | *nifU* | 1.15 | an assembly factor for iron-sulfur culsters |
| *sll0621* | *ccdA* | 3.09 | putative c-type cytochrome biogenesis protein |
| *slr0513* | *futA2* | 0.59 | iron transport system substrate-binding protein |
| *sll0247* | *isiA* | 0.33 | iron-stress chlorophyll-binding protein |
| *slr1204* | *htrA* | 3.21 | protease |
| sll1135 |  | 1.35 | hypothetical protein |
