# Supplementary material for: Global Transcriptional Profiles of the Copper Responses in the Cyanobacterium Synechocystis sp. PCC 6803
Source: PLoS One. 2014 Sep 30;9(9):e108912. doi: 10.1371/journal.pone.0108912 (PMC4182526; doi:10.1371/journal.pone.0108912)
Supplement: Table S7 — Oligonucleotides used in this work. (DOCX) [file pone.0108912.s015.docx]

Table S7. Oligonucleotides used in this work.

| **Name** | **Sequence** |
| --- | --- |
| **PetEF** | ACAATCCTCGCTGGCCTTCT |
| **PetER** | CGACAACTTTGCCTACCATG |
| **copM1F** | AGCATTCCCATGGGTAATCAATTCTGGATAT |
| **copM1R** | CATCTCGAGTCACTGACCATACCAGTTTTGATA |
| **slr2015F** | CGCTAGAACTAGGGCAGT |
| **slr2015R** | GACATTGAGATCTTCGTCC |
| **slr1667F** | CTGTGTCAGTGAGTTCGG |
| **slr1667R** | TTGCTCTTCTGCGATTGC |
| **sufRF** | CACGTCTTCACTCAGACCAC |
| **sufRR** | GTATTCAACGACAGCCAACT |
| **sufBF** | GTCAACCAACCCTACAAA |
| **sufBR** | ATGTTCCTGCAATGCTTC |
| **copBF** | AAGTCAATCGGCTCAGTCTG |
| **copBR** | GTAAACGACTTTGGGTTCTC |
| **petJF** | ATTCAACCAAGCTAGCCGAA |
| **petJR** | TCCGCTTGATCAAGCACGTA |
| **csoR5_Left** | TGGTTCCACGAGTACGATTG |
| **csoR5_Right** | ATGAATTTTACCCGGATTGACCGGTTTCAACCGACAATGCTC |
| **csoR3_Left** | TTATTTCAGTATGTTATCACACGGTTTTTACAACCTGTCGCC |
| **csoR3'_Right** | CTCGGCGGAGACTATCTTTG |
| **ARSB2** | GGATGTGAATGGAACAATGGGG |
| **ARSB3** | CAACGGGGGTCTAGCAAAGTGG |
| **NIA3** | CCCAATTTGAGGTGGTGTGATG |
| **NIA4** | ATAAAGGCAAGGGCTAGAGCAG |
| **NRP1** | CCCATATGGGCAAACTACCGCCTATC |
| **NRP3** | CCCAGGAAATAAACAGACAATCCCC |
| **∆copM1** | CGCTAGGTTGAAGGCTCTTG |
| **∆copS4** | CACTCTCTACAGTCAAAACAC |
